# Supplementary material for: Are carbon emissions trading and green financial instruments synergistic? -Comprehensive quantitative research based on content analysis
Source: PLoS One. 2024 Mar 7;19(3):e0298601. doi: 10.1371/journal.pone.0298601 (PMC10919601; doi:10.1371/journal.pone.0298601)
Supplement: S1 Data — (ZIP) [file pone.0298601.s001.zip › Data/地级市Z_F工作报告.zip!/║╙─╧/╓▄┐┌╩╨/└┤╫╘╣1⁄2╓┌║┼í╛┬φ┐╦╩2╛▌═°í┐_2010.docx]

各位代表：

　　现在，我代表市人民政府，向大会报告政府工作，请予审议，并请各位政协委员和其他列席人士提出意见。

　　一、2009年工作回顾

　　2009年是我市经济社会发展进程中极不平凡的一年，也是我市在危机中寻求机遇，在逆境中奋力追赶，在挑战中加快发展的重要一年。面对复杂多变的形势，在省委、省政府和市委的正确领导下，市人民政府始终坚持以科学发展观为统领，团结带领全市人民，千方百计保增长，全力以赴保民生，不遗余力保稳定，较好完成了市二届人大四次会议确定的各项目标任务，经济社会保持了平稳较快发展态势。

　　——经济总量跃上新台阶。初步核算，全市生产总值1111.7亿元，增长12%，高于全省平均增幅1.3个百分点。其中，第一产业增加值319.4亿元，增长4.3%；第二产业增加值483.6亿元，增长13.2%；第三产业增加值308.7亿元，增长17.6%。主要经济指标增幅高于全省平均水平，缩小了与全省平均水平的差距。

　　——粮食生产再创新高。粮食总产143.3亿斤，增长1.5%，总量和增量均居全省第1位，连续6年丰产丰收，被评为全省粮食生产先进市。

　　——工业经济快速发展。规模以上工业增加值287.4亿元，增长17.7%，高于全省平均增幅3.1个百分点；利润总额124.5亿元，增长27.4%，质量效益明显好转。工业投资255亿元，增长37.5%，工业发展后劲进一步增强。

　　——财政收支大幅增长。财政一般预算收入30.9亿元，增长21.1%。其中，市本级一般预算收入7.9亿元，增长24.9%。税收收入21.2亿元，增长18.7%。其中，市本级税收收入5.6亿元，增长19.6%。上述4项指标增幅均居全省第1位；财政一般预算支出164.1亿元，增长27.4%，支出总量居全省第4位。

　　——金融运行态势良好。金融机构各项存款余额792.5亿元，较年初增加128.8亿元，增长19.4%；各项贷款余额499.8亿元，较年初增加104.6亿元，增长26.5%，成为全省贷款增量过百亿的8个市之一。

　　——市场贸易繁荣活跃。社会消费品零售总额413.6亿元，增长20.2%，增幅居全省第2位。外贸进出口总额3.2亿美元，其中出口1.37亿美元，增长11.5%，是全省5个实现增长的省辖市之一。

　　——民生得到持续改善。城镇新增就业人员8.9万人，下岗失业人员再就业4万人，困难人员再就业1.5万人，城镇登记失业率控制在3.85%，动态消除“零就业家庭”。城镇居民人均可支配收入11363.3元，农民人均纯收入3907.8元，分别实际增长10.9%和8.1%。就医、就学、住房和社会保障等关系群众切身利益的问题得到有效缓解。

　　——社会保持和谐稳定。及时化解人民内部矛盾，妥善处置各类突发事件，严厉打击违法犯罪，平安周口建设成效明显，安全生产形势总体平稳，被评为全省安全生产优秀单位。

　　一年来,我们突出抓了以下重点工作：

　　(一)坚持项目带动增后劲。面对金融危机带来的影响，坚持项目带动战略不动摇，紧紧围绕中央新增投资项目、省重点项目、市100个重点项目和“双十五工程”，优化项目建设环境，完善项目协调推进机制和联审联批制度，开展重点项目观摩点评活动，推进投融资平台建设，保持了投资对经济增长的持续拉动。全社会固定资产投资690.6亿元，增长29.2%，高于省定目标9.2个百分点。争取中央新增1000亿元、1300亿元、700亿元资金项目572个，完成投资23.3亿元，项目开工率和地方财政配套资金落实率均为百分之百，居全省先进行列。争取中央新增800亿元资金项目77个，完成投资6193.6万元，达到了序时进度要求；列入省“8511”项目13个、总投资67.2亿元，“1074”重大工业结构调整项目44个、总投资76.6亿元，均完成了年度投资计划，被省政府表彰为投资目标完成先进单位；100个市重点项目投资73.2亿元，基本完成年度建设任务。“双十五工程”中的漯阜铁路(周口段)电气化改造、雨润集团300万头生猪屠宰加工等项目进展顺利；益海粮油日榨油1000吨生产线、亚华汽车安全玻璃生产线、财鑫多元醇生产线、裕达公司豆制品加工等项目竣工投产。沙颍河、涡河近期治理工程全面完成，顺利通过国家淮委会验收。

　　(二)坚持抓好“三农”强基础。面对历史罕见的冬春连旱等自然灾害，采取有效措施，积极应对。投资8.9亿元，改造中低产田24.6万亩、高标准农田建设示范工程项目1万亩，新增有效灌溉面积78万亩，粮食生产在大灾之年实现了丰产丰收，粮食总产分别占全省和全国的13.3%和1.4%，为国家粮食安全作出了新的贡献。淮阳、鹿邑、太康、商水被评为全国粮食生产先进县，项城、西华、郸城、沈丘被评为全省粮食生产先进县。继续深入开展“畜牧业发展推进年”活动，发展大中型规模养殖场区1248个，肉、蛋、奶产量分别增长8.4%、14.2%和16.5%。扎实推进林业生态市建设，我市被命名为“国家现代林业建设示范市”和“绿化模范城市”，全年实现林业总产值61.8亿元，增长20%。大力推广农业机械化作业，耕种收综合机械化水平达到72%。进一步改善农村生产生活条件,新改建农村公路1662公里；新增户用沼气8万户；新建改造农家店1700多家；解决44.8万人饮水安全问题；实施农民健身工程286个；新完成150个贫困村整村推进扶贫任务，解决和巩固温饱贫困人口20万人。发展农民专业合作社1433家，总量居全省第2位。农村土地流转92.3万亩，占耕地面积的7.6%。全面实施“111”工程，新农村建设试点工作实现良好开端。

　　(三)坚持服务企业上水平。建立“企业服务年”联席办公制度和市县领导定点联系帮扶100家企业制度，累计为企业解决各类问题422个。积极组织银企对接，帮助企业融资170.5亿元。强化产销衔接，积极开拓市场，加大对困难行业和企业的帮扶力度，企业活力显著增强。新增规模以上工业企业247家，总数达到970家。全部工业增加值429.5亿元，增长12.3%；工业用电量23.4亿千瓦时，增长10%。8家企业荣获“河南省优秀民营企业”称号，9家企业荣获“河南省高成长型民营企业”称号。

　　(四)坚持扩容提质抓城建。进一步完善城乡规划，完成中心城区港口物流园区、沙南老城区的控制性规划，以及9个县市的城市总体规划修编和省下达的乡镇、村庄规划任务。加大基础设施投入，城镇固定资产投资494.1亿元，增长30.4%，高于省定目标7.4个百分点。城镇建成区面积新增18平方公里，城镇人口新增22万人。城镇化率达到29.49%，提高1.84个百分点。继续实施中心城市带动战略，开工建设或拓宽改造东新区周口大道、文昌大道、天一路等8条道路，完成市体育馆、科技馆、文化艺术中心主体工程，周口卫校新校区、科技职业学院新校区等项目建设进展顺利；开发区基础设施进一步完善，一批项目入驻开工，承载能力和发展活力进一步增强；稳步实施老城区改造，建设路贾鲁河桥建成通车。改造供水低压区14处，新打深水井20眼，新增供水能力3.6万吨。硬化小街小巷191条，亮化主次干道7条，完成西大街低洼区除涝改造，建成6座垃圾中转站和12座公厕，新增天然气用户1.2万户，城市功能日益完善。加强城市精细化管理，开展中心城区环境综合整治活动，市民生活环境得到改善。扎实推进县城和小城镇建设，84个建制镇的生活垃圾处理设施建设任务全部完成。城镇承载能力和辐射带动能力明显提升。

　　(五)坚持转变方式谋发展。充分利用金融危机形成的倒逼机制，大力推进结构调整和产业升级，经济转型步伐明显加快。扎实推进产业集聚区建设。采取政府主导、社会投资、市场运作等多种方式，融资10亿元信贷资金，用于支持产业集聚区建设。11个产业集聚区规划全部通过省政府评审，累计投资96亿元，新开工项目122个，投产项目56个，建成区面积37.7平方公里，产业集聚效应初步显现。加快推进企业自主创新和战略重组。实施31项省级以上科技项目，科技创新能力得到加强。新建省级工程技术研究中心和企业技术中心7家、市级工程技术研究中心18家，2家企业被评定为省高新技术企业，金丹乳酸企业技术中心升级为国家级企业技术中心。通过合并、兼并等方式，25家企业与国内知名企业实施了重组，45家企业进行了技术改造。鼓励、支持、引导非公有制经济发展。全市新增私营企业1964户、个体工商户1.8万户。非公有制经济占GDP比重达到60.6%，高于省定目标0.6个百分点。加快发展服务业。在巩固提升商贸、餐饮、娱乐、运输等传统服务业的同时，加快文化旅游、金融保险、现代物流、信息服务等现代服务业发展。全年旅游人数920万人次，增长20.7%；实现综合性收入42.3亿元，增长21.7%。我市被命名为“中国优秀旅游城市”，淮阳龙湖被确定为国家湿地公园。保险行业保费收入41.5亿元，增长39.8%，居全省第4位；理赔7.8亿元，增长21.2%，居全省第7位。黄淮农产品物流大市场等现代物流项目开工建设。加强节能减排和生态环境建设。关停小火电23.2万千瓦，更换节能灯具7.6万台。境内6条出境河流水质断面COD平均达标率为96.5%，氨氮平均达标率为76%，达到了省定目标要求；COD新增削减量7040吨，超额完成任务。二氧化硫新增削减量3500吨，提前完成“十一五”减排任务。

　　(六)坚持改革创新增活力。加快投融资体制改革，大力扶持金融业发展，出台《关于支持我市金融业发展的意见》，设立中小企业贷款风险补偿基金，金融生态环境明显改善，国有控股银行不良贷款率大幅下降，市城市信用社成功过渡为商业银行，金融机构支持地方发展能力不断增强。积极搭建投融资平台，组建市综合投资公司、农业综合开发公司等5大投资公司，融资能力不断提高；组建中小企业担保公司21家，融资担保体系初步形成。深化文化体制改革，推进文化旅游融合发展，完成淮阳文化改革发展试验区总体规划编制，组建文化产业投资有限公司和文化旅游产业担保中心，融资32.6亿元用于项目开发建设。全面创新文化事业发展和文化惠民新模式，电影《胡辣汤》、《太昊陵》正式开拍，“周末一元剧场”公益性文化品牌被中宣部向全国推广。继续推进行政审批制度改革，市直14个职能部门成立行政审批科入驻市行政服务中心，初步构建了高效、便民的行政审批新机制。顺利启动市、县政府机构改革，医药卫生体制改革、人事制度等各项改革迈出了新步伐。创新招商引资理念和方式，有针对性地搞好产业和项目对接，累计新增省外资金项目249个，实际到位资金151.9亿元，增长21.6%。实际利用外资1.1亿美元，增长7.2%。我市被省政府表彰为招商引资工作先进单位。

　　(七)坚持改善民生促和谐。用于改善民生的财政支出81.6亿元，占财政总支出的49.7%，事关民生的“十大实事”和中心城区10件实事全部完成。积极做好就业再就业工作。实施“万人创业计划”，落实创业引导资金1598万元，发放小额担保贷款1.1亿元，带动8.7万人就业。实施“阳光工程”等培训计划，培训农村劳动力10.1万人，新增转移就业农村劳动力19.9万人。高校应届毕业生就业率继续保持较高水平。完善社会保障制度。企业养老保险、失业保险、城镇职工基本医疗保险、城镇居民医疗保险和生育保险参保人数，均有所增加。扶沟县新型农村养老保险试点工作成效明显，16周岁以上农村居民参保率90%以上，得到省政府充分肯定。全面实行新型农村合作医疗制度，提前完成国家3年内农村居民全覆盖目标，沈丘县在上海建立了全市第一家域外新农合定点医院。积极缓解困难群体住房难题，开工建设中心城区12万平方米、县(市)8.6万平方米经济适用住房。建成川汇区社会保障性住房629套、廉租住房283套，补贴低收入家庭廉租房租金285万元。关心低收入群体基本生活，为5.3万城市低保户和23.4万农村低保户分别发放低保金2.2亿元和2.9亿元，向城市低保群众发放价格补贴150万元。大力发展慈善事业，募集资金3974.7万元用于扶贫济困。全面发展社会事业。大力发展教育事业，不断提升办学水平，全市高招上线率突破50%，被清华、北大录取37人，创历史新高。积极开展全民健身活动，竞技体育水平不断提升，沈丘籍运动员张志磊在全国十一届运动会上获得拳击男子 91公斤级冠军。繁荣文化艺术事业，建设33个乡镇综合文化站和260个农家书屋，新建文化信息资源共享工程5个县级支中心和1288个村级基层点，送戏下乡演出2941场，为农村公益放映电影5.7万场；《都市长虹》、《云锦人家》等一批优秀剧目获省级优秀成果奖。积极改善群众医疗卫生条件，建设标准化卫生室1500个，改造乡镇卫生院39个，市中医院荣升三级甲等中医院。甲型H1N1流感、手足口病、艾滋病、结核病等传染病防治防控工作成效显著。积极开展创建“白内障无障碍市”活动，为2.5万名白内障患者实施了免费手术。完善计划生育利益导向机制，人口自然增长率控制在4.15%。，项城市、鹿邑县荣获全国计划生育优质服务先进县(市)。食品药品监管体系建设进一步加强，我市被评为全省首批食品安全优秀城市。扎实有效开展“家电下乡工程”，家电下乡产品销售和财政补贴兑付均居全省前列。审计、监察、统计、物价、气象、人防、档案、史志、广播电视、外事侨务、爱国卫生等工作也取得了新成绩。援助四川省江油市大堰乡、西屏乡恢复重建工作进展顺利。

　　(八)坚持加强精神文明和民主法制建设保稳定。深入开展“全民共铸诚信”活动。积极推进文明城市创建工作，38个单位被评为省级文明单位。加强文化市场管理，推进新闻出版业健康发展，“扫黄打非”斗争取得新成效。“五五”普法深入开展，法律援助工作成效显著。自觉接受人大法律监督、工作监督和政协民主监督，办理人大代表建议98件、政协委员提案405件。密切联系工会、共青团、妇联等人民团体，广泛听取社会各界意见和建议。全面贯彻实施行政许可法、行政复议法等法律法规，法治政府建设和依法行政工作扎实推进。加强政府廉政建设，反腐败斗争取得新成效。深入开展国防教育和双拥共建活动，吉鸿昌将军纪念馆被命名为全国国防教育示范基地，我市及鹿邑县、沈丘县、西华县被命名为省级双拥模范城(县)，军政军民团结进一步巩固。深入开展民族团结进步教育活动，民族宗教工作得到进一步加强，我市被省委、省政府命名为河南省民族团结进步先进单位，沈丘县、淮阳县分别被国务院和省政府表彰为全国和全省民族团结进步县。广泛开展“安全生产年”活动，各类事故起数、死亡人数分别下降26.5%、28.9%。精心组织开展严打整治斗争，不断完善社会治安防控体系，人民群众安全感明显增强。建立健全应急机制，认真排查化解矛盾纠纷，社会大局保持稳定。

　　各位代表，在困难和挑战面前，我市经济社会保持了平稳较快发展，呈现出追赶跨越的好趋势、好态势、好气势，这是全市上下和衷共济、共克时艰的结果，凝聚着全市人民的心血和智慧，得益于社会各界的大力支持和帮助。在此，我代表市人民政府，向工作在全市各条战线上的工人、农民、知识分子、各级干部、离退休老同志、人民解放军指战员、武警消防官兵、公安司法干警，致以崇高的敬意！向给予政府工作大力支持的人大代表和政协委员，向各民主党派、工商联、无党派、各人民团体和各族、各界人士，向驻周单位及所有关心、支持我市现代化建设的同志们、朋友们，表示衷心感谢！

　　回顾一年来的工作，我们清醒地认识到，尽管我们取得了明显进步，但就总体发展水平而言，周口依然处于全省落后位置，我市人口多、基础差、底子薄、人均发展水平低的现状还没有根本改变。经济社会发展仍然面临着许多困难和问题：一是经济结构不合理，发展方式粗放，产业层次不高，工业主导作用不强，第三产业发展滞后，结构调整难度较大。二是一些地方和部门，拼抢意识和责任心不强，对事关全局的工作和重点项目谋划不够，抓具体、抓深入的措施不力，项目储备不足，前期工作和基础性工作不扎实，个别项目建设没有达到预期目标。三是城市规划建设管理水平不高，事关民生的城乡基础设施落后，历史欠账较多，民生改善任务繁重。四是有些政府部门存在着思想解放不够、行政效率不高、工作落实不力等问题；个别工作人员官僚主义、形式主义、以权谋私、贪污腐败现象仍然存在。对此，我们一定高度重视，采取有效措施，认真加以解决。

　　二、2010年政府工作的总体要求和奋斗目标

　　各位代表，今年是实施“十一五”规划的最后一年，也是巩固经济回升基础、谋划“十二五”发展、加快追赶跨越的关键之年。我们既面临重大机遇，又面对严峻挑战。我们要正确认清形势，坚定必胜信心，自觉、准确地把握好发展的阶段性特征，趋利避害，打破常规，积极作为，奋力赶超，努力谱写经济社会发展的新篇章。

　　今年政府工作的总体要求是：全面贯彻党的十七大和十七届三中、四中全会精神，坚持以科学发展观为指导，认真落实中央、省委和市委经济工作会议各项决策部署，按照“重在持续、重在提升、重在统筹、重在为民”的要求，以经济建设为中心，以和谐稳定为重心，以项目建设和民生工程为抓手，大力实施招商引资和开放带动战略，强力推进工业化、城镇化和农业现代化，积极推进各项改革和体制机制创新，保增长、扩内需、调结构、促转型、惠民生、保稳定，努力实现经济社会平稳较快发展。

　　经济社会发展的主要预期目标是：生产总值增长11 %；地方财政一般预算收入增长12%；全社会固定资产投资增长22%；社会消费品零售总额增长16%；出口总额增长11%；实际利用外商直接投资增长15%；居民消费价格涨幅3%左右；城镇居民人均可支配收入实际增长7.5%，农民人均纯收入实际增长7%；人口自然增长率控制在6.5%。以内；城镇新增就业6万人，城镇登记失业率控制在4.5%以内；单位生产总值能耗降低2.5%，化学需氧量、二氧化硫排放量分别下降3.38%和1.7%；发展方式转变和经济结构调整取得重大进展。

　　各位代表，按照上述总体要求和预期目标，做好今年政府工作，需要着力把握好以下原则：一是必须坚持科学发展不动摇，遵循发展规律，贯彻市委要求，尊重群众意愿，防止大轰大嗡、大起大落，不走弯路，始终掌握经济工作主动权；二是必须坚持保增长、调结构、促转型不动摇，把保增长与调结构、促转型结合起来，努力实现速度与结构、质量、效益相统一，增强发展的可持续性；三是必须坚持项目带动不动摇，大抓项目、抓大项目，以项目带动“三化”协调发展，增强发展后劲；四是必须坚持品牌带动不动摇，强化品牌意识，注重品牌创建，做大做强工业品牌，培育壮大劳务品牌，全力打造文化品牌，形成品牌效应；五是必须坚持创新带动不动摇，深化改革，扩大开放，不断推动体制机制创新，增强发展的动力和活力；六是必须坚持服务带动不动摇，进一步转变作风、提高效能，少提口号、多干实事，说到做到、说好做好；七是必须坚持改善民生不动摇，以人为本、重在为民，认真解决关系人民群众切身利益的突出问题。

　　各位代表，周口还是一个欠发达地区，面对激烈的市场竞争，追赶跨越的任务十分繁重。如果我们稍有懈怠，就会错失良机、落于人后。我们必须进一步强化使命意识、机遇意识和竞争意识，万众一心、团结拼搏，抓住机遇、乘势而上，以新思维、新思路、新举措谋求工作新突破。虽然前进道路充满坎坷，但实践将会证明，任何艰难险阻都改变不了我们创造美好幸福生活的强烈愿望，任何危机挑战都阻挡不了我们加快追赶跨越的坚定步伐。

　　三、千方百计保持经济社会平稳较快发展

　　保持全市经济社会平稳较快发展，必须统筹谋划，重点突破，紧紧抓住牵动全局的重点工作，强力推进。

　　(一)强力推进项目建设，保持投资较快增长

　　今年我市围绕国家和省投资重点，筛选建设项目100个，总投资227亿元。在整体推进的同时，突出抓好事关经济社会发展、具有重大支撑和带动作用的“双十五工程”。

　　——突出抓好15项重点工业项目。15项重点工业项目，总投资69.46亿元，今年投资30.73亿元。

　　1.总投资3亿元，今年完成全部投资，开工并建成金丹乳酸年产10万吨高品质乳酸项目；

　　2.总投资2.1亿元，今年完成全部投资，开工并建成辅仁药业年产150亿粒(片)固体药项目；

　　3.总投资1.8亿元，今年完成全部投资，开工并建成泓宇纺织项目；

　　4.总投资3.5亿元，今年投资1亿元，开工建设长城门业年产200万樘防盗门项目；

　　5.总投资1.9亿元，今年投资1.1亿元，开工建设平康电气系列高压电气设备项目；

　　6.总投资1.5亿元，今年投资8000万元，开工建设华丰网业高强多层造纸网项目；

　　7.总投资27亿元，今年投资2亿元，继续建设隆达热电厂2×30万千瓦机组项目；

　　8.总投资8亿元，今年投资4亿元，继续建设大用集团西华分公司肉鸡产业化项目；

　　9.总投资2.5亿元，今年投资1亿元，继续建设佳利达纺织项目；

　　10.总投资2亿元，今年投资1亿元，继续建设蜀中制药技改扩建项目；

　　11.总投资5.6亿元，今年完成剩余投资5.12亿元，建成宏亿木业中高密度纤维板项目；

　　12.总投资4亿元，今年完成剩余投资2.8亿元，建成景鹏肉类加工项目；

　　13.总投资2.56亿元，今年完成剩余投资1.86亿元，建成龙源纸业三期工程；

　　14.总投资2.3亿元，今年完成剩余投资1.5亿元，建成通泰纺织10万纱锭项目；

　　15.总投资1.7亿元，今年完成剩余投资1.65亿元，建成豫人木业高密度纤维板扩建项目。

　　——突出抓好15项重点基础设施项目。15项重点基础设施项目，总投资61.9亿元，今年投资32.07亿元。

　　1.总投资8.79亿元，今年投资4亿元，开工建设郸城至淮阳一级公路改建项目；

　　2.总投资1.4亿元，今年投资4600万元，开工建设市幼儿师范学校；

　　3.总投资1.15亿元，今年投资2000万元，开工建设市中心医院门诊楼；

　　4.总投资1.1亿元，今年投资6800万元，开工建设市广播电视多功能发射塔；

　　5.总投资3615万元，今年完成全部投资，开工并建成市东新区110千伏输变电工程；

　　6.总投资34亿元，今年投资20亿元，继续建设漯阜铁路(周口段)电气化改造项目；

　　7.总投资4.77亿元，今年投资1亿元，继续建设涡河(周口段)航运开发一期项目；

　　8.总投资9000万元，今年投资6000万元，继续建设市体育场；

　　9.总投资9500万元，今年投资5000万元，继续建设市科技职业学院新校区；

　　10.总投资1.99亿元，今年完成剩余投资7952万元，建成市沙南污水处理厂二期工程项目；

　　11.总投资1.8亿元，今年完成剩余投资5000万元，建成周口大道拓宽改造工程项目；

　　12.总投资1.7亿元，今年完成剩余投资1.4亿元，建成市五一路改造工程项目；

　　13.总投资1.22亿元，今年完成剩余投资3200万元，建成大庆路桥改造工程项目；

　　14.总投资1.21亿元，今年完成剩余投资6900万元，建成周口卫校新校区；

　　15.总投资5629万元，今年完成全部投资，建成市人民路改造工程项目。

　　“双十五工程”是政府推进项目建设的重要抓手，市里将出台具体扶持政策，有关县市区和政府工作部门要在用地、融资、用电等要素供给方面给予优先考虑，严格项目责任，优化建设环境，为项目建设提供优质、高效、便捷的服务。

　　(二)强力推进工业发展，提升工业经济质量和效益

　　加大企业服务帮扶力度，把产业做大、企业做强和品牌做优结合起来，加快构建优势突出、富有竞争力的工业主导产业体系，加速推进工业经济向更大规模、更高层次发展。

　　实施“企业服务行动计划”。进一步完善“领导分包制”、“现场办公”、“一企一策”、“银企对接”等做法，将服务重点转向促进转型升级、战略重组和开拓市场等方面，切实为企业解难题、办实事、谋出路、促发展。建立健全政策导向、市场开拓、融资服务、困难企业帮扶等长效机制，提高行政效能，推动企业服务工作常态化。认真落实各项优惠政策，减轻企业负担，优化企业发展环境。加强对重点行业、骨干企业和主要产品的市场监测分析，坚持月度运行监测与季度分析制度，做好分析、研判、预测和预警，及时出台相应的对策和措施，保持工业经济快速发展。

　　培育和壮大骨干企业。以编制“十二五”规划为契机，完善工业发展规划，进一步明确骨干企业和支柱产业发展目标和措施。支持30户重点企业和10户高成长性企业与国内外500强、同行业100强联姻结亲，吸纳优势资源，实现规模扩张。鼓励益海粮油、财鑫集团、辅仁集团等行业龙头企业兼并困难企业，推进关联产业、上下游企业融合重组，力争完成10家以上企业联合重组，争取新增一批销售收入超10亿元的企业。继续抓好企业上市培育，支持金丹乳酸、金丝猴集团等企业上市直接融资。同时，鼓励支持中小企业走小而专、小而精道路，指导帮助中小企业改进工艺装备，淘汰落后产能，搞好科技创新，优化产品结构，实现上档升级，争取规模以上工业企业突破1000家，形成以大型企业为龙头、骨干企业为支撑、中小企业为基础，主导产业突出、关联产业互补的发展格局。

　　加快产业集聚区建设。认真贯彻落实我市《关于加快产业集聚区科学发展的意见》，积极整合资源、提升功能、强化特色、增强竞争力。继续完善并实施11个产业集聚区各项规划，加快产业集聚区基础设施建设和体制机制创新，健全产业集聚区投融资、中小企业担保、土地开发整理等机制，提升市场化运作能力。全年力争完成各类投资150亿元。积极培育食品加工、纺织服装和医药化工等支柱产业，巩固发展电力、机械、化工等基础产业和网业、尾毛等特色产业。设立园区内企业专项扶持资金，实行重大项目优惠政策，进一步优化投资环境。大力开展招商引资，积极承接产业转移，加快园区组装步伐，每个产业集聚区力争新上亿元以上项目2-3个，尽快形成一批辐射带动能力强、特色主导产业突出的产业集群和产业基地。

　　增强企业自主创新能力。全面落实自主创新体系建设规划，积极实施“自主创新体系建设工程”，鼓励天豫经贸、金丹乳酸、华丰网业等企业积极参与国家标准制定。继续加强与中科院、清华大学、郑州大学等科研院校的科技合作，加快构建企业为主体、市场为导向、产学研相结合的技术创新体系。推进工程技术研究中心、企业技术中心等自主创新平台建设，力争新建省级工程技术研究中心1-2家、市级工程技术研究中心10家。实施“质量兴市”战略，落实好市长质量奖励制度。推动企业产品结构调整，提高产品的技术含量和附加值。加强品牌培育，争取新增省名牌5个、著名商标10个，进一步提升工业产品的知名度。

　　(三)强力推进新农村建设，促进农民持续增收

　　坚持把解决“三农”问题作为重中之重，以促进农民增收为根本目的，强化对“三农”的支持，加快推进农业现代化进程。

　　进一步稳定和提高粮食综合生产能力。认真贯彻落实中央一号文件精神，积极组织实施《国家粮食战略工程河南核心区建设规划》和省“粮食稳产保收行动计划”，粮食播种面积稳定在1600万亩。大力优化品种结构，着力提高粮食单产和品质，确保粮食总产稳定在145亿斤左右。抓好大型商品粮基地和土地整理与复垦开发等项目建设，强化科技支撑和高产示范，搞好农业科技创新，重点抓好小麦、玉米、大豆等“高产创建万亩示范区”，切实发挥示范带动作用。加强防洪排涝工程建设，加大中小河流治理力度，投资2亿元用于病险涵闸除险加固和小型农田水利建设，抓好沈丘槐店灌区节水改造续建和平原洼地治理工程，提高防汛抗旱能力。加大农业综合开发力度,实施中低产田改造项目10万亩、高标准农田建设示范工程项目4万亩。加快推进农业机械化，继续提升农机装备水平和服务能力。加强重大农业气象灾害监测、预警、预报工作，提高农业应对极端天气气候能力。认真落实强农惠农政策，保护和调动农民种粮积极性。

　　调整结构壮大特色产业。大力发展无公害农业、品牌农业和观光休闲农业，稳定发展棉花、油料、食用菌、优质特色蔬菜等经济作物，因地制宜发展林果业。继续开展“畜牧业发展推进年”活动，依托大用邦杰、陈州华英、雨润集团、志元食品、金丝猴集团等龙头企业，大力推进规模养殖和标准化生产，新建标准化养殖场(小区)180个以上。完善动物疫病防控体系，进一步提高疫病监测防控能力。支持农产品加工业发展，积极推进农业产业化经营，健全农产品销售网络，打造周口农产品品牌。推广“龙头企业 农民专业合作组织 农户”的产业化经营模式，重点抓好“50家龙头企业培育工程”、“50个标准化生产基地建设工程”和“50家示范性农民专业合作社扶持工程”，培育农业产业化龙头企业集群，力争市级以上重点龙头企业发展到220家。

　　拓宽渠道促进农民增收。充分挖掘农业内部增收潜力，鼓励农民发展多种经营，创办“短平快”增收项目，通过股份合作参与兴办农业资源开发及二三产业项目。坚持就地转移、异地输出与自主创业一起抓，发挥各类中介组织和经纪人作用，分门别类搞好技能培训和配套服务，提高农民工组织化程度，实现劳务输出280万人次，增加农民非农收入。建立严格规范的直补渠道，确保各类农业补贴资金直接发放到农民手中。有效控制农资价格，继续清理涉农收费，切实加强农民负担监督管理，严防农民负担反弹。

　　继续改善农村生产生活条件。深入实施“111”工程，重点抓好20个省级示范性中心村建设，每个县市区开工建设或建成1-2个新型农村住宅社区。进一步完善农村基础设施，解决28万农民饮水安全问题，完成省定沼气建设任务。推进农村骨干路网建设，实施“乡乡连、县县畅”工程，改建农村公路400公里、大中危桥36座。加强县乡公路超载超限治理，加大农村公路管护力度，确保农村公路完好畅通。继续实施“万村千乡”市场工程，新建440个农村连锁超市，覆盖全市85%以上行政村。加快农村邮政网络建设，补扩建22个空白乡镇邮政局(所)，新建1500个行政村“村邮站”。

　　创新制度推进农村改革。稳定和完善农村基本经营制度，加强土地承包经营权流转管理和服务，在全市10个县市区和1/3的乡镇建立起流转服务大厅，鼓励农民在依法自愿有偿流转的基础上，发展多种形式的适度规模经营。抓好商水、淮阳、西华3个全国基层农技推广改革与建设示范县各项改革措施的落实，争取其余7个县市区跨入全国示范县行列。大力发展农民专业合作社，积极发展农业农村各种社会化服务组织，不断提高农业生产经营组织化程度。加快农村金融体制创新，着力推进农村信用社改革，积极培育村镇银行、贷款公司、农村资金互助社和小额贷款组织。推动小型农田水利设施产权制度改革，全面完成集体林地林权登记发证工作。

　　(四)强力推进城镇化进程，统筹城乡协调发展

　　坚持以紧凑型、复合型理念规划建设城市，优化空间布局，完善城市功能，促进产城融合，增强城镇吸纳和辐射带动能力，力争城镇化率提高1.8个百分点。

　　高标准编制城乡规划。坚持规划先导，用科学规划引领发展。按照省“现代城镇体系建设工程”要求，结合漯阜铁路电气化改造，搞好周口市总体规划调整，完善中心城区控制性详细规划，加快县城控制性详细规划编制。在2月底前、6月底前和年底前3个时间段，分别完成省定的村庄布局、村镇体系、乡镇总体和中心村(社区)的规划编制，用规划指导新农村建设。

　　增强中心城市综合承载能力。提升各类投融资平台融资能力，放大政府投资集聚吸纳金融资本、社会资本、外来资本投入城市建设的“乘数效应”，更多地运用BT、BOT模式推进重大工程项目建设运营。完善中心城区道路、供水、供电、燃气、污水和垃圾处理等基础设施，提高承载能力。加快开发东新区。抓好安居小区、申凤梅艺术中心、人防指挥中心等项目建设，启动张寨、康店“城中村”改造试点工程，推进农信社、移动公司、中国电信等项目建设。组装完善开发区。按照打造工业基地、聚集城市产业的要求，抓住漯阜铁路电气化改造、新火车站广场建设的机遇，进一步提升开发区的吸引力和竞争力，争取更多客商投资兴业。积极改造老城区。着眼于改善环境、完善功能、提升品位，加大城市排水系统改造力度，抓好沙北污水处理配套管网建设。加快部分棚户区改造，落实城市拆迁各项政策措施，严格按规定标准及时兑现补偿。实施亮化美化绿化工程，亮化次干道5条，新建垃圾中转站5个，新建改造公厕10座，新增公共绿地100万平方米。

　　建立城市管理长效机制。结合市、县政府机构改革，继续完善“两级政府、三级管理、四级网络”的城市管理机制，充分发挥川汇区、开发区和东新区的作用，分层次落实城市管理主体责任，全面推行街道社区网格化管理和居民自我管理。切实强化公共设施维护管理，组织开展行政综合执法和道路交通专项整治。以创建省级卫生城市为载体，实施城市清洁工程，落实好“门前五包”责任制，突出抓好城市出入口、城乡结合部及公共聚集场所、小街小巷的集中整治，实现净、亮、美，为广大居民营造良好的居住环境。

　　抓好县城和小城镇扩容提质。进一步完善中小城镇供水、供电、污水和垃圾处理等基础设施及医院、学校等公共设施，增强城镇对经济发展和人口集聚的综合承载能力。把城市发展和产业培育结合起来，推动产城互动，以建设产业集聚区为平台，以发展特色主导产业为重点，着力培育一批有支撑带动能力的城区工业企业，壮大县域经济，促进农民就近就地就业，逐步把县城发展成为20万以上人口的中等城市，把39个国家、省、市重点小城镇发展成为区域中心城镇。

　　推进城乡统筹发展。建立城乡统一体制，健全以城带乡、以工促农机制，优化各种资源要素在城乡间的配置，推进土地集中、产业集中、人口集中。加快国家公路运输枢纽城市建设，开工建设周口客运总站，推进8个县级客运站建设，进一步完善公路、铁路、水路三位一体的综合交通运输体系。加快户籍管理、社会保障制度改革，规范企业用工行为，切实保障农民工权益，逐步实现农民工在劳动报酬、子女就学、公共卫生、住房租购以及社会保障方面与城镇居民享有同等待遇；采取有针对性的措施，着力解决新生代农民工问题。积极稳妥地开展以土地承包经营权置换社会保障、以宅基地置换城镇房产试点工作，促进在城镇稳定就业和居住的农民有序转变为城镇居民。

　　(五)强力推进经济结构调整，加快转变发展方式

　　把保持经济平稳较快发展和加快转变发展方式有机统一起来，在发展中促转变，在转变中谋发展。

　　促进产业结构优化升级。以省里实施十大产业振兴规划和“转型升级双千工程”为契机，抓好我市51个省级重大工业结构调整项目、市级33个技术改造项目，加快推进产业结构优化升级，着力构建符合我市实际的现代产业体系。改造提升传统产业。积极采用先进适用技术改造和提升食品加工、纺织服装和医药化工三大支柱产业，积极引导项城市、鹿邑县、郸城县、沈丘县和市本级的食品加工企业，扶沟县、项城市、商水县的纺织服装生产企业，鹿邑县、郸城县、川汇区的医药化工企业，太康县的锅炉产业，淮阳县的塑编产业，加快技术改造，不断提升产品竞争力和市场占有率。积极培育战略先导产业。以电子信息、生物科技等产业领域为重点，以科信电缆、阳光电缆和久通电缆等企业为依托，打造周口电力、通讯电缆光缆生产基地；以金丹乳酸、辅仁集团、远东生物等生物科技企业为重点，加快高新技术产业化，打造生物产业生产基地，培育新的经济增长点。加快发展服务业。巩固提升商贸、餐饮、娱乐、运输等就业容量大的传统服务业，加快发展教育培训、社区家政、中介服务、信息服务等成长性强的现代服务业，努力形成优势互补、协调发展的新格局，不断提高服务业在国民经济中的比重。切实抓好港口物流园区、黄淮农产品物流大市场、扶沟雨润农副产品物流配送中心等重点项目，加快发展现代物流业。进一步优化金融生态环境，鼓励金融保险机构创新业务，推进信用工程创建，促进金融保险业健康发展。扩大城乡居民消费需求。加强企业工资支付保障制度建设，积极稳妥推进义务教育学校、公共卫生、基层医疗卫生事业单位实行绩效工资，严格落实最低工资制度，继续提高企业退休人员基本养老金、部分优抚对象待遇、城乡居民最低生活保障和农村五保户供养水平，增强居民消费能力。继续实施“万村千乡”市场工程、“双百”市场工程和农村超市对接工程，落实“汽车下乡”、“家电下乡”等优惠政策。加快宽带网络建设和农村信息化步伐，促进信息消费。整顿和规范市场秩序，维护好消费者合法权益，营造良好的消费环境。

　　加强节能减排和生态环境建设。完善节能减排体制机制，进一步提高资源利用率，推动节约集约发展。坚持节约集约用地。切实履行政府耕地保护特别是基本农田保护职责，加大土地执法监察力度，严肃查处土地违法案件。严格建设用地批后监管，探索建立用地绩效评价机制，不断提高投资强度。大力开展土地“三项整治”，加快盘活存量土地，落实城乡建设用地增减挂钩政策，确保重大项目用地需求。健全土地开发整理中心，推进中心城区新增建设用地及耕地占补平衡，确保中心城市的发展需要。抓好节能降耗。大力推广清洁生产，发展低碳经济、绿色经济和循环经济。抓好太康县国家再生能源建筑应用示范县、市经济开发区国家太阳能光电建筑应用项目。强化建筑节能管理，民用建筑节能设计执行率达到100%。支持发展和应用新型墙材。加强环境保护。依法关闭不符合国家产业政策、污染严重的企业，坚决淘汰落后技术、设备和产品。完善污水处理厂污水管网配套，提高污水处理率，平均运行负荷达到80%以上。

　　抓好运粮河污染综合治理和食品、医药、化工等氨氮排放量较大行业的环境综合整治。继续加强农村环保，建立完善“以奖代补”、“以奖促治”机制，建成86个乡生活垃圾中转站，搞好畜禽养殖、水产养殖、秸秆综合利用和土壤污染防治，控制农村面源污染。推进生态文明建设。强化对水源、土地等自然资源的生态保护，加快现代林业示范市建设和淮阳龙湖国家湿地公园建设，加强农田林网、城镇绿化、廊道绿化等林业生态工程建设，完成植树造林2600万株、66.7万亩，完成商水、项城林业生态县创建和500个村镇绿化工程，年内创建50个市级生态文明村、5个环境优美小城镇，全面完成林业生态市创建任务，提升我市绿化整体水平。

　　(六)强力推进文化建设，实现文化旅游融合发展

　　坚持文化旅游两篇文章一起做，把文化、旅游产业作为扩大消费、调整结构的重要内容，着力培育新的经济增长点。

　　大力发展文化事业。鼓励支持文艺创作，着力打造一批具有周口特色的文艺精品。大力实施文化惠民工程，建成27个乡镇文化站、338个农家书屋、5个达到国家标准的文化馆信息共享工程县级支中心、2810个村级服务点。继续实施舞台艺术送农民和农村电影放映工程，为全市所有乡镇免费送一场演出，为每村每月公益性放映一场电影。加大“扫黄打非”力度，强化网络文化的建设与管理。加快市文化艺术中心等文化设施建设，抓好市县两级公共图书馆、文化馆、博物馆建设。加强重点文物和非物质文化遗产保护和开发利用。

　　着力发展文化产业。全面落实文化产业扶持政策，加快书画古玩市场等文化产业资源要素整合，搞好汝阳刘笔业开发，着力推进演艺业等文化产业发展和关帝庙文化产业园区建设。加快淮阳文化改革发展试验区建设步伐。进一步打造提升公益性文化品牌“周末一元剧场”。支持发展多媒体广播电视、网络广播影视、数字多媒体广播、手机报等新兴文化产业，积极推动文化娱乐、休闲健身和艺术培训等社会文化产业发展。

　　加快发展旅游业。加强景区建设，重点开发太昊陵、老子故里、关帝庙、女娲城、吉鸿昌将军纪念馆、袁世凯故里、叶氏庄园等景区，扶持发展中原民俗文化园等景区，注重提高景区内涵和品位。加快改革创新，推动资源整合，组建旅游集团，不断增强市场竞争力。加大对外宣传推介力度，加强同国内外旅行社和知名旅游企业的联系与合作，不断提高周口旅游的知名度。积极实施旅游富民工程，在全市范围内扶持发展5个旅游村和400户农家乐，开展乡村旅游，推动旅游向更深层次、更大范围发展，提升旅游产业发展的综合效益。

　　(七)强力推进改革创新，全方位扩大开放

　　认真解决制约科学发展、追赶跨越的深层次问题，把改革创新和对外开放推向深入，不断增强经济社会发展活力。

　　突出抓好重点领域改革。继续深化国企改革，推进国企战略重组，重点抓好流通企业改革。大力发展非公有制经济，鼓励、支持、引导更多的民营资本进入基础设施建设、公共事业、金融服务、社会事业等领域。深化财税体制改革，进一步挖掘税源潜力，实现财税收入稳定增长，增加对教育、卫生、科技等社会事业支出，推进基本公共服务均等化。加快推进文化体制改革，全面推进电台、电视台制播分离，完成市县文化市场综合执法改革任务。稳步推进医药卫生体制五项改革，完善基本医疗保障制度。积极稳妥地推进市县政府机构改革和事业单位分类改革，深化各项人事制度改革，开展公益性事业单位法人治理结构改革试点。推动收入分配、资源价格、环保收费等领域改革，深化水利、科技等行业管理体制改革，进一步激发发展活力。

　　大力开展招商引资。坚持专业化、市场化、园区化、产业化招商引资的路子，深入开展大招商活动，继续实行县(市、区)政府和市直主要经济部门招商引资目标管理责任制，严格兑现奖惩。优化招商引资项目库，增强项目投资吸引力。积极参加省政府组织的各类经贸合作洽谈会等重大经贸活动，集中发布项目，强势推介周口。加大工业、基础设施和服务业项目的招商力度，加强与央企、省企合作，不断拓宽引资领域。紧紧抓住当前东部沿海地区产业加速向中西部地区转移的有利时机，有针对性地搞好产业承接，争取更多、更大、更好的项目落户我市，完成实际利用省外资金172亿元、增长15%的目标。

　　积极扩大外贸出口。抓住全省实施“对外开放行动计划”的机遇，继续做好外贸、外资、外经工作，推动外向型经济又好又快发展。加强对莲花味精、金丹乳酸、三闸纺织等重点企业和重点进出口商品的监控和跟踪服务，及时了解和帮助解决重点企业生产中存在的困难和问题，保持重点企业进出口持续稳定增长。落实鼓励出口的政策措施，加强对郸城财鑫糖业、扶沟力神机械、西华凯鸿鞋业、商水强鸿鞋业等具有出口潜力企业的服务引导，尽快扩大出口规模，形成新的外向型经济增长点。

　　四、切实改善民生，促进社会和谐

　　认真办好“十大民生工程”，切实关心百姓的衣食住行、安危冷暖，注重解决涉及群众切身利益的问题，高度重视维护社会和谐稳定。

　　(一)千方百计扩大就业。落实省“就业促进行动计划”，继续开展全民创业，鼓励发展就业容量较大的服务业、劳动密集型产业，以及个体私营和中小企业，多渠道、多方式创造更多的就业岗位，以创业带动就业，确保全市城镇失业人员再就业3万人，其中帮助“4050”人员等就业困难群众就业1万人，动态消除“零就业家庭”。加强对高校毕业生就业指导，做好复转军人就业安置工作。加大创业帮扶工作力度，扩大小额担保贷款发放范围，发放贷款1.5亿元。继续实施“阳光工程”、扶贫培训“雨露计划”和“农村劳动力技能培训计划”，落实农民工回乡创业的各项政策，促进农村劳动力转移就业、回乡创业，新增转移就业农村劳动力15万人。

　　(二)健全城乡社会保障体系。完善城乡低保制度，适当提高城乡低保标准，将符合低保条件的困难群众全部纳入覆盖范围，做到应保尽保。逐步建立和完善临时救助机制。继续提高企业退休人员养老金，幅度不低于全市平均养老金水平的10%。制定完善养老保险关系转移接续办法，继续抓好扶沟县新型农村社会养老保险试点。完善落实被征地农民补偿机制和社会保障制度。继续扩大社会保险覆盖范围。基本解决各类关闭破产企业退休人员和困难企业职工医疗保障、养老保险问题，基本实现工伤保险市级统筹。加大保障性住房建设力度，开工建设经济适用房17万平方米，为城市低收入住房困难家庭提供3300套经济适用住房。加大廉租住房租赁补贴发放力度，年内补贴1.2万户。加快农村危房改造，探索城镇廉租房租售并举政策。健全城乡医疗救助制度，将城镇居民医保和新农合财政补助标准提高到每人每年120元。完善城乡社会救助体系，大力发展残疾人事业、社会福利和慈善事业，保障和改善困难群众的基本生活。加大扶贫开发力度，解决17万农村贫困人口脱贫问题，完成120个扶贫开发重点村整村推进任务。

　　(三)积极发展各项社会事业。千方百计加大各项社会事业投入力度。优先发展教育事业，落实“人力资源素质提升行动计划”，努力把人口压力转化为人力资源优势。加快教育信息化建设，完善学校尤其是农村学校布局调整，推进城乡教育质量均衡发展，努力解决进城务工人员随迁子女入学和中心城市大班额问题。开工建设东新区中学，建成投用东新区实验幼儿园。扎实推进中小学校舍安全工程建设，继续落实各项义务教育优惠政策。大力发展职业教育，实施职业教育攻坚计划，重点建设一批示范性职业院校和职业教育实训基地。加强中医药建设，投入使用市中医院门诊病房综合楼。健全城乡医疗卫生服务体系，建设1000个行政村标准化卫生室、改建两所县级医院(中医院)、10所中心乡镇卫生院和3所城市社区卫生服务中心。进一步做好甲型H1N1流感等重大疾病防控工作，切实维护人民群众生命安全。整顿规范医疗市场，严厉打击非法行医。扩大新农合门诊统筹试点，积极推进新农合就地直补，大病住院报销封顶线提高到6万元。提高城镇职工、城镇居民基本医保最高支付限额标准，城镇职工大病报销封顶线提高到13.6万元，城镇居民大病报销封顶线提高到6.6万元。加快市体育场馆等体育设施建设，继续实施农民体育健身工程、全民健身工程。强化人均意识，抓好计划生育工作，稳定低生育水平。

　　(四)进一步加强精神文明建设。深入开展学习实践科学发展观活动，加强社会主义核心价值体系建设，大力弘扬以爱国主义为核心的民族精神和以改革创新为核心的时代精神。在城市深入开展以优美环境、优良秩序、优质服务，创建文明城市、创建文明社区、创建文明景区为主要内容的“三优三创”竞赛活动，着力提高市民文明素质和城市文明程度；在农村以文明乡镇创建和文明卫生村户评选为载体，深入开展“清洁家园行动”活动，改善村容村貌。积极推进殡葬改革，引导农民移风易俗。以打造诚信周口为目标，以“知荣辱、讲正气、促和谐”道德实践活动为载体，深入开展“共铸诚信”活动。进一步组织好文化、科技、法律、卫生“四下乡”和科技、文体、法律、卫生“四进社区”活动，提高全民科学文化素质。

　　(五)全力维护社会和谐稳定。全面启动安全周口创建活动，加强安全生产源头治理，继续强化烟花爆竹等重点行业治理整顿，开展道路交通和消防安全专项治理，遏制较大事故发生。继续开展食品药品安全专项整治，提高食品监督抽查合格率。认真做好信访稳定工作，引导群众依法有序表达诉求，积极化解社会矛盾。深入推进社会管理创新、公正廉洁执法，妥善处理土地征用、拆迁安置、企业改制、环境污染、劳资纠纷、医患纠纷、涉法涉诉等方面的矛盾和问题，有效预防和妥善处置群体性事件和突发性公共事件。继续推进平安周口建设，搞好困难群体法律援助工作。积极健全社会治安防控体系，依法防范和严厉打击“两抢一盗”等各类违法犯罪活动，确保社会大局和谐稳定。

　　同时，继续加强国防教育，提高全民国防意识。支持驻周部队和民兵预备役建设，继续做好人民防空工作，广泛开展省级双拥模范城(县)创建活动，进一步密切军政军民关系。深入开展促进民族团结进步活动，巩固和发展平等团结、互助和谐的社会主义民族关系。全面贯彻党的宗教工作基本方针，发挥宗教界人士和信教群众在促进经济社会发展中的积极作用。充分发挥各民主党派、工商联、无党派人士的参政议政作用，积极支持工会、共青团、妇联等群众团体开展工作。认真做好第六次人口普查工作。努力做好统计、气象、地震、档案、保密、外事、侨务、史志等工作。完成四川省江油市大堰乡、西屏乡援建任务。全面完成“十一五”规划各项目标任务。编制好“十二五”规划。

　　五、加强政府自身建设

　　新形势新任务对政府工作提出了新的更高要求。各级政府工作人员要加强学习、增长才干，把握大局、注重运作，求真务实、开拓创新，严格自律、廉洁勤政，更好地担负起加快周口发展的历史重任。

　　(一)转变工作作风。紧紧围绕“两转两提”、“提速增效”，大兴真抓实干、雷厉风行的工作作风，坚决反对形式主义、官僚主义，反对假大空，反对哗众取宠。建立健全抓落实的领导机制和工作机制，对市委重大决策和部署，明责任、抓到位、见效果，严格执行纪律，加强督促检查，做到政令畅通、令行禁止。大力整治文风会风，开短会，发短文，讲短话，严格控制各类检查、评比，务实有效地举办会展和节庆活动，腾出更多精力谋大事、谋发展，集中更多时间抓具体、抓落实。继续广泛开展民主评议市直单位及其重点科室(二级机构)活动，严肃查处涉企“四乱”行为，进一步优化经济发展环境。

　　(二)提高行政效能。把管理创新与优质服务结合起来，改革政府管理体制，理顺政府部门分工和权责关系，强化协调联动，形成推动工作的整体合力。进一步减少和规范行政审批事项，健全岗位责任、限时办结、服务承诺等制度，加强和改进“一站式”服务，将行政服务中心真正建设成为集政务服务、信息公开、效能监督于一体的综合服务平台。深化政务公开，强化效能监察，加强电子政务和行政效能电子监察系统建设。完善政府绩效评估体系和行政问责制度，坚决杜绝政府权力部门化、部门权力利益化，大力整治行政不作为、乱作为、办事拖拉、效率低下等问题，不断提高政府办事效率和服务水平。

　　(三)坚持依法行政。进一步强化法制意识，认真贯彻落实《国务院关于加强市县依法行政的决定》和各项法律法规，加强行政执法队伍建设，提高依法施政水平，努力做到行政合法、程序正当、高效便民、诚实守信。全面实施依法行政工作目标责任制，进一步健全完善执法责任、错案追究、举报投诉等制度，确保权力依法公正运行，确保权力始终受到监督。坚持向人大报告工作和向政协通报工作制度，认真执行人大及其常委会的决议、决定，自觉接受人大法律监督、工作监督和政协民主监督，认真办理人大代表议案、建议和政协委员提案。深入开展普法教育，提高全社会自觉学法守法用法意识，夯实社会和谐的法治基础。

　　(四)建设廉洁政府。加强惩治和预防腐败体系建设，深入开展警示教育和岗位廉政教育，认真落实党员领导干部廉洁从政若干准则。加强对重点领域和关键岗位权力运行的监督，坚持用制度管权、管事、管人，严格执行领导干部问责制、廉政承诺制、行政执法责任制和经济责任审计制。加强对财政资金、重大投资项目的审计和监察。严肃查处违纪违法案件，坚决纠正侵害群众利益的不正之风。全体政府工作人员特别是领导干部一定要做到警钟长鸣、秉公用权、廉洁自律，争做清廉型干部，建设廉洁型政府。

　　各位代表！

　　形势催人奋进，事业任重道远，发展时不我待，责任重于泰山。新的一年里，我们一定倍加珍惜难得的历史机遇，倍加珍惜来之不易的发展态势，倍加珍惜团结和谐的大好局面，把人民的期望当作前进的动力，把崇高的使命化为积极的行动，在市委的坚强领导下，紧紧依靠全市人民，励精图治，锐意进取，扎实工作，为建设繁荣富裕、文明开放、稳定和谐的新周口而努力奋斗！
